# You eat what you are: personality‐dependent filial cannibalism in a fish with paternal care

**DOI:** 10.1002/ece3.1966

**Published:** 2016-01-29

**Authors:** Martin Vallon, Christina Grom, Nadine Kalb, Dennis Sprenger, Nils Anthes, Kai Lindström, Katja U. Heubel

**Affiliations:** ^1^ Animal Evolutionary Ecology groupUniversity of Tübingen72076TübingenGermany; ^2^ Environmental and Marine BiologyÅbo Akademi University20520TurkuFinland

**Keywords:** Behavioral syndrome, individual variation, infanticide, parental care, spillover effect

## Abstract

Many animal parents invest heavily to ensure offspring survival, yet some eventually consume some or all of their very own young. This so‐called filial cannibalism is known from a wide range of taxa, but its adaptive benefit remains largely unclear. The extent to which parents cannibalize their broods varies substantially not only between species, but also between individuals, indicating that intrinsic behavioral differences, or animal personalities, might constitute a relevant proximate trigger for filial cannibalism. Using a marine fish with extensive paternal care, the common goby (*Pomatoschistus microps*)*,* we investigated the influence of animal personality on filial cannibalism by assessing (1) behavioral consistency across a breeding and a nonbreeding context; (2) correlations between different breeding (egg fanning; filial cannibalism) and nonbreeding (activity) behaviors, and, in a separate experiment; (3) whether previously established personality scores affect filial cannibalism levels. We found consistent individual differences in activity across contexts. Partial filial cannibalism was independent of egg fanning but correlated strongly with activity, where active males cannibalized more eggs than less active males. This pattern was strong initially but vanished as the breeding season progressed. The incidence of whole clutch filial cannibalism increased with activity and clutch size. Our findings indicate that filial cannibalism cannot generally be adjusted independently of male personality and is thus phenotypically less plastic than typically assumed. The present work stresses the multidimensional interaction between animal personality, individual plasticity and the environment in shaping filial cannibalism.

## Introduction

Parental care is a common phenomenon widespread throughout the animal kingdom (Gross and Sargent 1985; Clutton‐Brock 1991; Royle et al. 2012). It incorporates any form of investment directed toward offspring that increases their survival (Clutton‐Brock 1991; Smith and Wootton 1995) and spans from the preparation of nests and the production of yolk‐containing eggs to directly provisioning, cleaning, and guarding eggs or young outside the parent's body (Trivers 1972; Blumer 1982; Clutton‐Brock 1991; Royle et al. 2012). Despite investing heavily in the survival of their progeny, parents in some species regularly eat their own young (Polis 1981; Klug and Bonsall 2007). This so‐called filial cannibalism (FC) occurs in a wide range of taxa (e.g., Anthony 2003; Gilbert et al. 2005; Miller and Zink 2012) and, notably, often co‐occurs with male brood care in teleost fishes (reviewed in Manica 2002b).

Although counterintuitive at first sight, FC is generally assumed to be adaptive by maximizing the cannibal's lifetime reproductive success (Rohwer 1978; Manica 2002b). The diverse array of hypotheses on adaptive functions of FC have recently been summarized by Klug and Bonsall (2007). One popular notion suggests that eggs or young provide an alternative food source, allowing parents to continue or improve care for the remaining current brood or enhance future reproduction (energy‐based hypothesis; Rohwer 1978; Sargent 1992). Some studies indeed found FC to decrease under better food availability or parental condition (e.g., Kvarnemo et al. 1998; Manica 2004), but this pattern is clearly not universal (e.g., Lindström and Sargent 1997; Klug and St Mary 2005; Klug et al. 2006). Other ideas such as selective cannibalism of inferior eggs (Klug and Lindström 2008) or a reduction of egg density to enhance oxygen availability to the remaining eggs (Payne et al. 2002, 2004) have also been challenged by contradictory results (e.g., DeWoody et al. 2001; Lissåker et al. 2003; Klug et al. 2006; Lissåker and Svensson 2008). Thus, given that all hypotheses either lack empirical evidence or have produced inconclusive results, filial cannibalism still constitutes an evolutionary conundrum (Klug and Bonsall 2007).

All current hypotheses on the adaptive function of FC share the (usually implicit) assumption that all individuals of a population respond in a similar way to changes in the environment. This in turn implies that most of the interindividual variation is determined through extrinsic factors. However, several studies found remarkable differences in cannibalistic behavior (e.g., with respect to the extent and time of egg consumption) between individuals under similar environmental conditions (e.g., Salfert and Moodie 1985; Nemtzov and Clark 1994; Lindström and Sargent 1997), hinting at intrinsic rather than extrinsic sources for some of the observed variation.

Intrinsic constraints on responses to environmental variation are captured in the concept of animal personalities, which characterizes the consistency in rank orders of individual behaviors over time and/or across contexts and thus quantifies limitations in responding to environmental change (Gosling 2001; Sih et al. 2004b; Réale et al. 2007). Recent work identified a broad range of personality traits (reviewed in Réale et al. 2007; Sih and Bell 2008; Stamps and Groothuis 2010), with at least some being heritable (Réale et al. 2007; Dochtermann et al. 2015; Petelle et al. 2015) and directly linked to reproductive success and survival (Smith and Blumstein 2008; Adriaenssens and Johnsson 2013; Mutzel et al. 2013). Consistent interindividual differences have been suggested to also occur in parental care behavior, inducing variance between parents in the effort attributed to their offspring (Roulin et al. 2010).

In the context of filial cannibalism, it is particularly relevant that personality traits may not vary in isolation. Instead, multiple behaviors often show population‐wide intrinsic correlations, or behavioral syndromes (sensu Sih et al. 2004a), with the boldness‐aggression syndrome in great tits being a classic example (Carere et al. 2005). This intrinsic linkage can lead to negative spillover effects, where individuals are limited to a certain behavioral type that is adaptive in some contexts but maladaptive in others (Sih et al. 2004a,b). For instance, Johnson and Sih (2005) found that in female fishing spiders, *Dolomedes triton,* high voracity levels in ontogeny are beneficial due to increased adult fecundity, but this aggressiveness spills over to adulthood, resulting in excessive precopulatory sexual cannibalism that massively depresses fitness. Limited behavioral plasticity due to behavioral syndromes could thus explain some peculiar behaviors (e.g., Sih et al. 2003; Johnson and Sih 2005).

We here pursue the idea that filial cannibalism, at least to some extent, may be the consequence of similar spillover effects from linked behaviors that are beneficial in different contexts. If true, this may also help to explain some of the contradictory results in previous experiments, because consistent individual differences may have confounded the experimental manipulation of extrinsic factors.

We thus investigated the influence of animal personality on filial cannibalism using the common goby (*Pomatoschistus microps,* Krøyer) as a model system. This benthic fish exhibits extensive paternal care (Nyman 1953) coupled with frequent filial cannibalism (Svensson et al. 1998). Spawnings occur readily in the laboratory and FC can easily be determined during an ongoing brood cycle (e.g., Kvarnemo et al. 1998; Jones and Reynolds 1999a), making *P. microps* an ideal system for studying animal personality in cannibalistic behavior. Specifically, we (1) investigated interindividual differences in *P. microps* using activity as a universal personality trait, repeatedly measured in different contexts; (2) checked for behavioral syndromes linking activity as a noncare trait with egg fanning as a representative paternal care trait and FC; and (3) tested whether fish allocated into groups based on previously established personality scores subsequently show predictable amounts of egg cannibalism.

## Materials and Methods

### Study species

**Figure 1:**
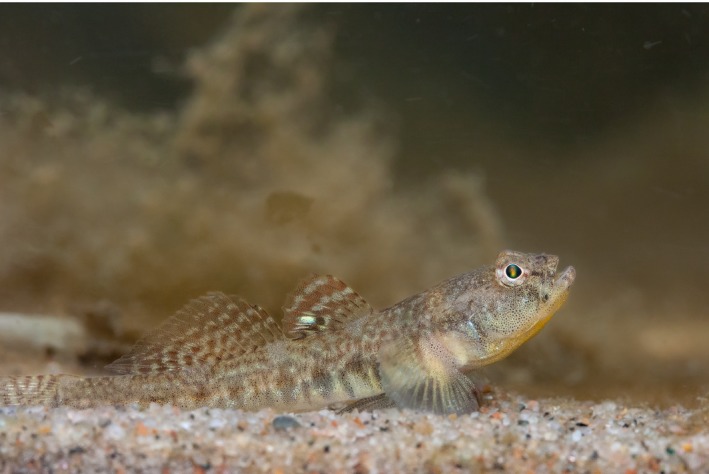
A male common goby (*Pomatoschistus microps*).

The common goby is a small, short‐lived fish that occurs abundantly in shallow soft‐bottom areas along the European coast, including the Baltic Sea. Reproduction takes place during several consecutive breeding cycles from May to August (Miller 1975). Males (Fig. 1) aggressively compete for suitable hard nest structures such as mussel shells and rocks (Borg et al. 2002) and try to actively lead females to their nest (Nyman 1953). After attaching the eggs to the ceiling of the nest, the female immediately abandons the clutch and leaves brood care entirely to the male, which guards, cleans, and ventilates the eggs (Nyman 1953). Males may accommodate clutches of several females simultaneously (Magnhagen and Vestergaard 1993). Offspring immediately desert the nest upon hatching 1 to 2 weeks later (Rogers 1988), then mature within 8–10 months, and usually die after a single breeding season in the following winter (Miller 1975).

### General setup

Two separate experiments were conducted in June and July 2011 and 2012, respectively, at Tvärminne Zoological Station near Hanko, Finland. Experiment 1 was designed to detect animal personality and behavioral syndromes in *P. microps* by assessing the candidate traits activity, egg fanning, and filial cannibalism (objectives 1 and 2). Experiment 2 tested whether males assigned to a high activity category showed different levels of FC than low‐activity males (objective 3).

Fish were collected close to the shoreline in a sheltered, sandy bay using a seine, except for males in 2011 caught at the same location from artificial nests (ceramic tiles measuring 5 × 5 cm) using a hand net while snorkeling. Following transfer to the wet laboratory, males were measured for body size (total length to the nearest mm) and placed into experimental aquaria, while females were housed in stock tanks prior to use. Each experimental tank contained sand as substrate and a halved flowerpot (4.5 cm diameter) placed upside down as artificial nest site. The ceiling of each nest was fitted with a plastic sheet onto which females laid their eggs during spawning. This sheet could be removed and reinserted without damaging the eggs, allowing repeated photography of individual clutches. The outside of each aquarium was covered with black plastic foil to prevent neighboring males from interacting. The front cover was temporarily removable to facilitate behavioral observations. All aquaria were continuously supplied with fresh seawater through a flow‐through system allowing natural temperature fluctuations. Males were fed daily with three large frozen chironomid larvae. Pretests indicated that this feeding regime was sufficient, as feeding larger amounts often resulted in leftover food. All fish were released into the wild after the experiments.

### Acclimatization period

Males were given 9 to 12 days to acclimatize to the aquarium environment and the experimental procedures to ensure normal behavior during the following testing period. In the first 5 to 6 days postcatching, fish were accustomed to feeding and direct behavioral observations with the front cover of the tank temporarily removed. After this initial acclimatization period, we gradually added more procedures in the following sequence: temporal removal of the front cover and behavioral observations from the front, water temperature measurements, briefly touching each artificial nest, and removal and reattachment of the plastic sheets in the artificial nests. Given that nest building – an important female choice criterion (Jones and Reynolds 1999b) – can be stimulated by the presence of females (Vestergaard 1976), we placed a female confined in a transparent Plexiglas container into each tank for 1 h on days seven and eight (Experiment 1). To exclude that fish may alter their behavior in the presence of observers in anticipation of food, we decoupled feeding and observations from day six onwards (Experiment 2).

### Experiment 1

Here, a behavior unrelated to brood care (activity) was measured repeatedly in a nonbreeding (males without clutch) and a breeding context (males caring for a clutch). While males were caring for a clutch, we additionally measured the two care‐related traits egg fanning and filial cannibalism.

#### Testing period

Thirty males were housed in individual 23‐L aquaria in a greenhouse under a natural day–night cycle. To control for order effects, individuals were assigned to two groups that differed in the sequence in which they were subjected to breeding and nonbreeding phases, that is Group 1 (mean ± SE total length: 35.2 ± 0.5 mm) started in the breeding phase while Group 2 (35.5 ± 0.5 mm) started in the nonbreeding phase.

Behavioral observations were carried out by a single observer from day 10 onwards for 25 days in total. Water temperature was measured daily before observations started. We then recorded activity during the nonbreeding phase and additionally egg fanning and filial cannibalism during the breeding phase (details below). Fish spent 3 days in each nonbreeding phase, while a breeding phase lasted for 5 days in total (at least 1 day for spawning and a maximum of 4 days for observations; see below). The two phases were repeated three times in an alternating manner for all males. On the first day of every breeding phase, one female was added to each tank at 13:00 h and removed the following morning at 09:00 h. Females were assigned to individual males according to body length. Although females were larger on average, this assured a similar male–female size difference among males (mean ± SE difference in total length: 1.9 ± 0.1 mm). Immediately upon female removal, we checked nests for the presence of eggs using a flashlight. Males without eggs received a new female at 13:00 h the same day and again the day after, but were not further considered during this particular breeding phase if still unsuccessful. If males cannibalized their whole clutch, this was recorded as total filial cannibalism (TFC) and corresponding fish were not observed anymore in this phase. Remaining clutches were removed at the end of each breeding phase. A total of 122 females were needed to provide enough spawning opportunities, all collected 4 days before each spawning event.

#### Measured behaviors

The order of observed tanks was randomized each day. However, we conducted the individual observations for each fish in the same order every day to improve comparability by keeping potential order‐related effects consistent across individuals (see Dingemanse et al. 2007, 2009).

Activity was measured as the number of pectoral movements resulting in a shift in spatial position of the male during 180 sec, independent of ground contact. During the breeding phases, we subtracted periods of egg guarding (i.e., male sitting in fanning position; see below) from the observation time span since fanning excludes behaviors that fall under our definition of activity.

Egg fanning, a paternal care behavior where males actively ventilate their eggs inside the nest using a fanning action of the fins (Nyman 1953), was recorded during activity measurements. The egg fanning rate was defined as the number of pectoral fin flaps per time the male spent in fanning position, that is sitting in the nest with the head facing outwards. Fish that did not show any egg fanning within this time span were observed for another 180 sec.

To quantify FC, plastic sheets with eggs were temporarily removed and photographed after the behavioral observations while being constantly submerged in seawater. Short‐term removal of egg clutches is a common method to determine the current number of eggs (e.g., Forsgren et al. 1996; Jones and Reynolds 1999a; Klug et al. 2006), and all males accepted the returned eggs without detectable changes in brood care behavior. Images were analyzed by manually counting eggs using the Cell Counter plugin (Kurt De Vos, University of Sheffield, UK) in ImageJ version 1.44p (Wayne Rasband, National Institutes of Health, USA). FC was defined as the number of eggs eaten per day. We followed the common notion that partial filial cannibalism (parents eat some of their offspring) and total FC (parents eat all of their current offspring) represent two distinct biological phenomena (proposed by Rohwer 1978) and analyzed cases of whole clutch FC separately.

#### Data analysis

We obtained repeated behavioral measurements for 23 males, of which 18 acquired a clutch in all three breeding phases. The remaining seven of the initial 30 males were excluded from statistical analysis.

Our primary goal in this experiment was to investigate the correlations within the same behavior across contexts as well as between different behaviors. In order to decompose raw phenotypic correlations into between‐ and within‐individual correlations (Dingemanse and Dochtermann 2013), we constructed multivariate generalized linear mixed‐effect models with four response variables – nonbreeding activity, breeding activity, egg fanning, and filial cannibalism (excluding TFC) – using the MCMCglmm package (Hadfield 2010) for R v. 3.0.3 (http://www.r-project.org). All behavioral response variables were best approximated by a Poisson error structure. We included *individual ID* as a random intercept to account for the repeated behavioral measurements and model between‐individual variation in mean response values. In addition, we included *context sequence* as a fixed factor and *clutch size* and *temperature* as covariates in the model. Backward model selection was based on the deviance information criterion (DIC), retaining factors only when their inclusion reduced the DIC by at least two (Spiegelhalter et al. 2002; Zuur et al. 2009; Arnold 2010).

Per definition, measurements of the predictor *clutch size* were available for the breeding phases only, requiring an initial reduced model without the response *nonbreeding activity*. Finding no contribution of *clutch size* to model fit, we removed this predictor and continued with a full model containing all four response variables. Further model selection rendered *context sequence* and *temperature* also uninformative, leaving a final model without fixed factors.

To assess the correlations between all response variables, we first retrieved the posterior distributions of between‐individual variances for each trait and between‐individual covariances between traits, calculated the correlations, and used the estimates of the corresponding posterior modes as correlation coefficients (see supplement of Dingemanse and Dochtermann 2013 for detailed procedure). We used 95 % Bayesian credible intervals for statistical inference (Gelman and Hill 2007; Kruschke 2014; Korner‐Nievergelt et al. 2015) as given by the highest posterior density (HPD) intervals for the acquired correlations (Dingemanse and Dochtermann 2013).

Throughout, we used standard noninformative priors (inverse Wishart) for multiple response variables. Changes in prior settings had only little effect on posterior distributions (data not shown). We achieved low levels of autocorrelation between successive samples by running each model for one million iterations with a 100,000 iteration burn‐in period and a thinning interval of 500. In addition to the main analysis, we investigated overall activity differences between contexts and the influence of other behaviors on the occurrence of TFC using standard methods in the R package lme4 (Bates et al. 2014).

### Experiment 2

**Figure 2:**
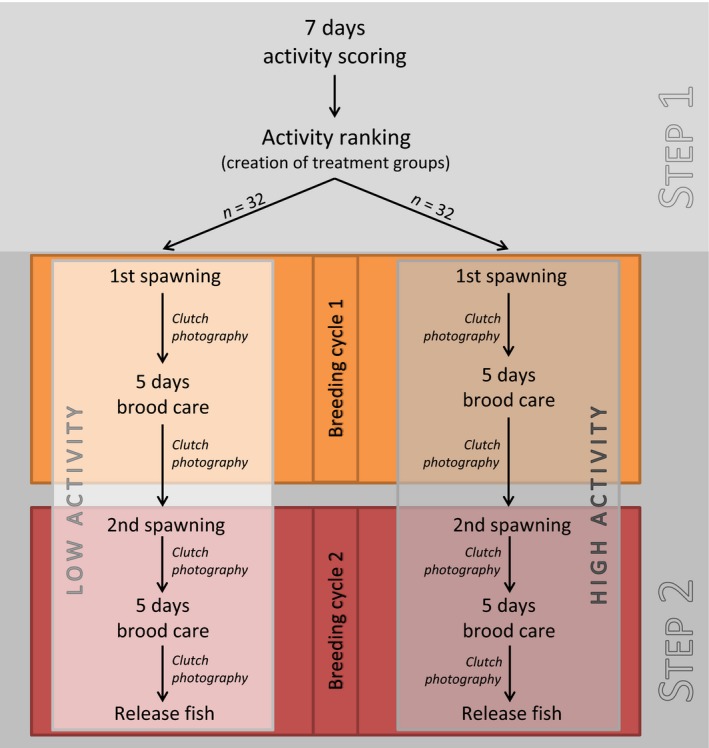
Schematic design of Experiment 2.

Given activity‐based personality established in Experiment 1, we here allocated males to a low‐activity and a high‐activity group and subjected them to two breeding cycles with clutches from two different females (Fig. 2). During this paternal care phase, we scored the amount of filial cannibalism exhibited by individual males, expecting more FC in high‐activity males.

#### Step 1: Activity scoring

One hundred and twelve male *P. microps* were individually housed in 5.8‐L plastic aquaria under a 16/8 h day/night light regime. Activity was scored as outlined for Experiment 1 in a randomized order twice daily over 7 days. Fish that developed infections or remained extremely anxious (*n* = 23) were excluded from the experiment and released. Average male activity showed significant individual consistency in the mean activity scores of the first and the last 2 days (Spearman rank correlation; *n* = 89, *ρ *= 0.52, *P *<* *0.001). Males were ranked by their average activity to create a low‐ and a high‐activity group (*n* = 32 each) after excluding fish with intermediate scores to maximize differences in activity levels (mean ± SE activity; low: 1.8 ± 0.2; high: 11.7 ± 1.6). Male body length did not differ between groups (*t*‐test; *t *=* *0.67, df = 62, *P *=* *0.503).

#### Step 2: Brood care

The 64 test males were randomly relocated into new individual tanks, spatially randomized over low‐ and high‐activity treatments. Fish experienced two consecutive phases of paternal care (5 days each), in which filial cannibalism was recorded. For spawning, one female was added to each male in the late afternoon and removed the next morning. Males that failed to spawn received another female for up to five times in the first and nine times in the second cycle. Females were size‐matched to males at ±2 mm with similar mean difference to male body size between groups (Wilcoxon test; *z* = 0.19, *n*_low_ = 53, *n*_high_ = 40, *P *=* *0.848).

To determine the amount of filial cannibalism (see Experiment 1), pictures of individual clutches were taken twice, on day one and on day five after spawning. We additionally measured water temperature twice per cycle (day two and four) to account for potential effects on cannibalistic behavior. Males were given 1 day to recover after the first phase until we added a new female for the second spawning.

#### Data analysis

We quantified cannibalism for 50 of initially 64 male common gobies, of which 44 spawned twice. To statistically compare cannibalism levels between low‐ and high‐activity males, we fitted a generalized linear mixed model (GLMM) with Poisson error distribution using the number of eggs eaten as response variable while excluding cases where males consumed their whole clutch. Total filial cannibalism was analyzed in a separate GLMM with binomial error distribution (TFC yes/no). Both models were specified as follows: We included *individual ID* and *spawning date* as random intercepts. Using random slopes for *individual ID* over the two breeding cycles was not possible because not all males provided data for both. Fixed factors included the assigned *activity* (low or high), *breeding cycle* (1 or 2), and their interaction. We further added *temperature*,* clutch size,* and *mean egg diameter* as covariates.

**Figure 3:**
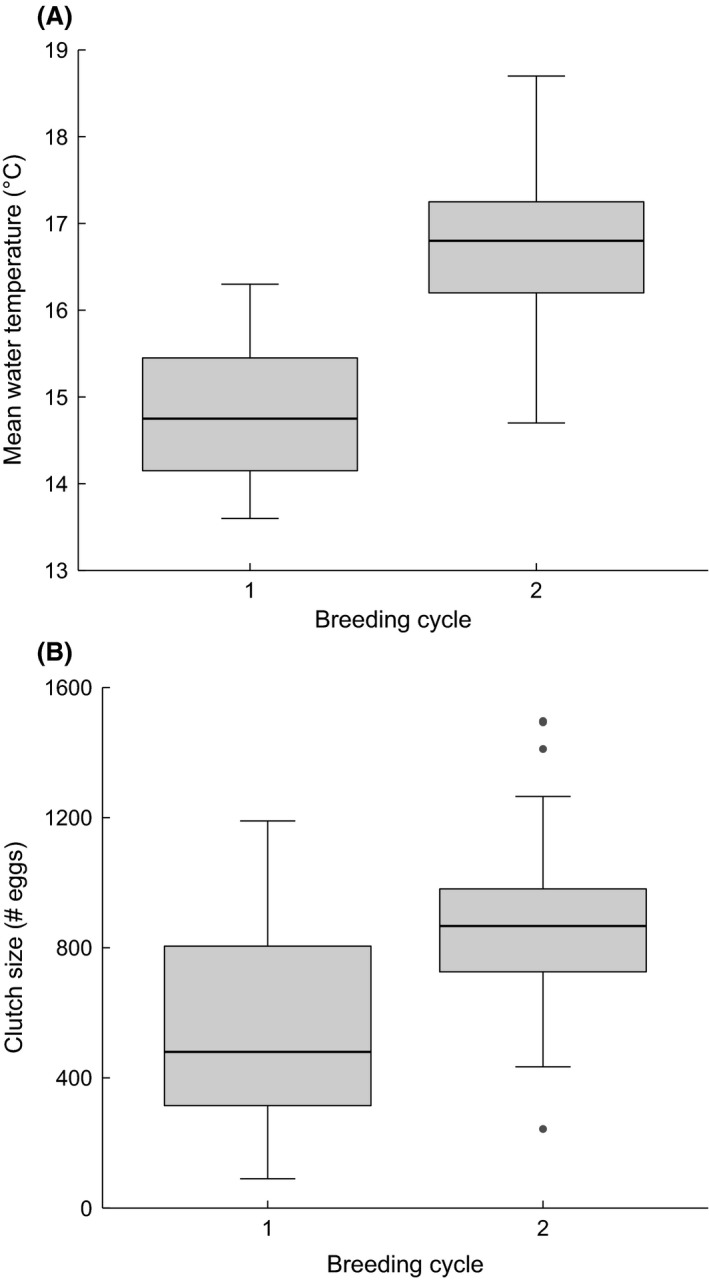
Differences between breeding cycles (*n*_1_ = 49, *n*_2_ = 45) in (A) mean water temperature per male and (B) clutch size.

There were large differences between breeding cycles in water *temperature* (Fig. 3A) and *clutch size* (Fig. 3B). To avoid confounding with *breeding cycle* in the model, we centered both covariates around their cycle means by subtracting the mean value for a respective cycle from each observation. In addition, to improve model convergence, *clutch size* was scaled by dividing each observation by the overall standard deviation and *mean egg diameter* was centered and scaled using the overall mean and standard deviation, respectively (Korner‐Nievergelt et al. 2015). We corrected for overdispersion in the Poisson model by including an observation‐level random factor (Gelman and Hill 2007; Korner‐Nievergelt et al. 2015). The Bayesian information criterion (BIC) was used to select the most parsimonious biologically meaningful model (Zuur et al. 2009). Neither *spawning date* nor *clutch size*,* temperature,* or *mean egg diameter* improved model fit. The final reduced model thus contained the fixed factors *activity* and *breeding cycle* and their interaction. All models were fitted using the lme4 package (Bates et al. 2014) in R v. 3.0.3 (http://www.r-project.org).

## Results

### Experiment 1

Average male activity differed significantly between nonbrood care and brood care contexts (*context z* = −9.22, *P *<* *0.001; GLMM with *individual* as random intercept, *context* and *context sequence* as fixed factors; *n *=* *346 observations of 23 individuals; no effect of *sequence*) with caring males being considerably less active (mean ± SE: 26.0 ± 1.3) than noncaring males (43.6 ± 1.3).

**Figure 4:**
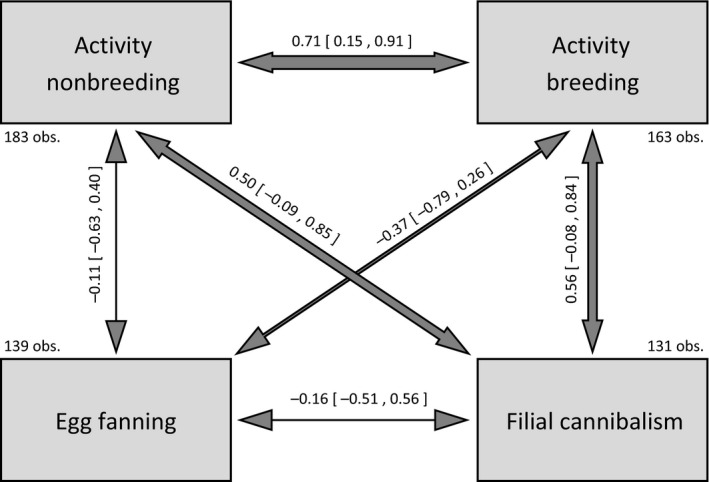
Behavioral correlations represented by correlation coefficients and their corresponding 95% credible intervals as estimated by a multiple‐response MCMC model (*n* = 131–183 observations of 23 individuals; number of observations for each behavior given in figure). Credible intervals not crossing zero identify significant correlations. Arrow thickness indicates the strength of the correlation.

Irrespective of these overall differences, males maintained their activity rank orders across both contexts, showing a positive correlation between breeding and nonbreeding activity (Fig. 4). In addition, activity in both contexts showed moderately strong and near‐significant correlations with filial cannibalism, with active individuals tending to consume more eggs (Fig. 4). In contrast, we found no behavioral syndromes linking the paternal care traits egg fanning and filial cannibalism, nor linking egg fanning and either activity measure (Fig. 4).

**Figure 5:**
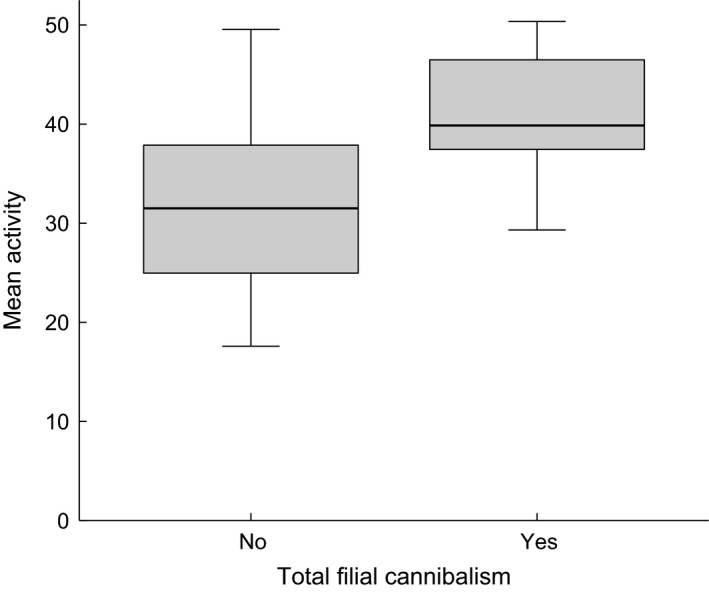
Difference in overall activity (mean of both contexts) between males that never showed total filial cannibalism (TFC_no_; *n* = 16) and males that fully cannibalized at least one of their clutches (TFC_yes_; *n* = 7).

In addition to covarying with partial filial cannibalism (PFC) as outlined above, activity predicted total filial cannibalism (TFC; binary logistic regression based on mean overall activity per individual; *z* = 2.03, *n*_no_ = 16, *n*_yes_ = 7, *P *=* *0.043). More active males were more likely to perform TFC (Fig. 5). No such relationship was found with egg fanning (*z* = 0.63, *P *=* *0.530).

### Experiment 2

**Figure 6:**
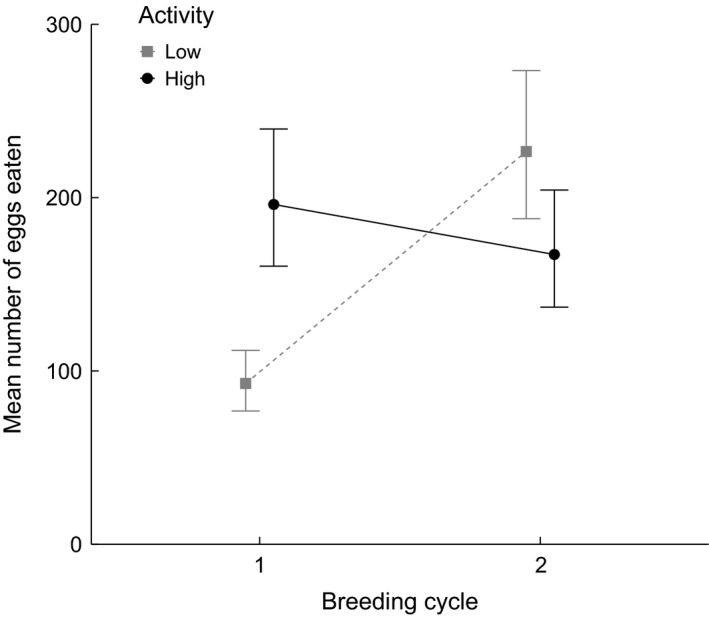
Interaction plot based on model estimates for the mean absolute number of eggs cannibalized per fish (excluding cases of TFC). Presented are group means and standard errors for each factor combination of activity and breeding cycle.

When assessing variation in partial filial cannibalism, we found a significant overall effect of *breeding cycle* on the number of eggs eaten, with more pronounced FC in cycle 2 (Table 1). In agreement with our main prediction, high‐activity males showed more cannibalism on average than low‐activity males (Table 1). However, these main effects were superseded by a significant interaction between *activity* and *breeding cycle* (Table 1; Fig. 6). A simple effects analysis revealed significantly more filial cannibalism by high‐ versus low‐activity males in cycle 1 (*z* = 2.20, *P *=* *0.028) but not in cycle 2 (*z* = −1.43, *P *=* *0.152).

**Table 1:** Fixed effect estimates from a generalized linear mixed model with Poisson error structure and individual as random effect. The model evaluated the effect of activity (low or high) and breeding cycle (1 or 2) on the number of eggs cannibalized by common goby males (*n* = 60 observations of 43 individuals). Note that estimates are on the log scale

	Estimate	SE	*z*‐value	*P*
Intercept	4.30	0.25	17.53	<0.001
Activity	0.81	0.37	2.20	0.028
Breeding cycle	1.16	0.36	3.20	0.001
Activity × Breeding cycle	−1.38	0.54	−2.55	0.011

**Figure 7:**
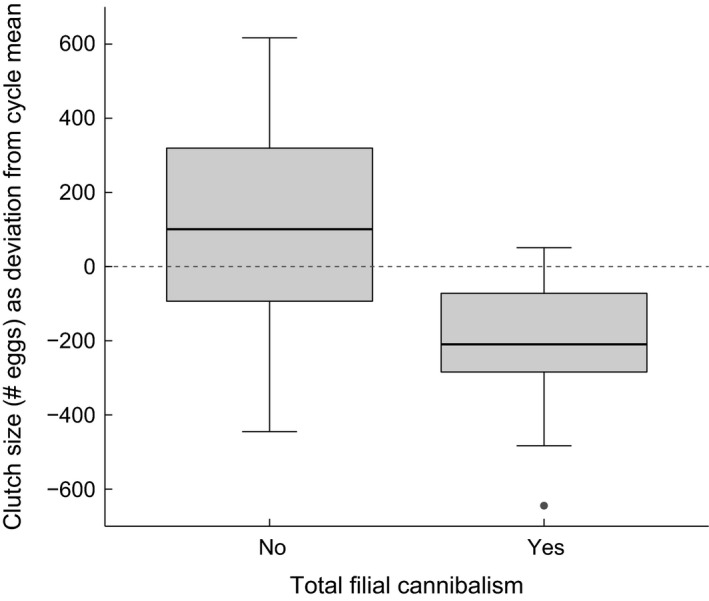
Difference in the original number of eggs between clutches that were fully cannibalized (TFC_yes_; *n* = 34) and clutches that were not (TFC_no_; *n* = 60). Values were centered on the mean of the corresponding breeding cycle, and deviations from zero thus indicate that clutches were larger or smaller than average within their cycle.

The final model for the analysis of total FC contained *activity* (maintained as our main experimental factor) and the covariate *clutch size*. Hence, in contrast to partial FC, TFC was unaffected by *breeding cycle*. Contrary to our predictions, *activity* did not affect the incidence of TFC (*z* = −0.37, *P *=* *0.714, *n *=* *94 observations of 50 individuals). Instead, TFC covaried with *clutch size* (*z* = −3.09, *P *=* *0.002), with males being more likely to consume the whole clutch when clutches were small (Fig. 7).

## Discussion

Using common gobies as a model system, we find – for the first time to our best knowledge – that animal personality predicts filial cannibalism. Males expressed consistent individual differences in general activity that remained stable over breeding and nonbreeding contexts. Moreover, activity formed a behavioral syndrome with filial cannibalism, and we experimentally show that more active males cannibalize more eggs than low‐active males. Contrary to earlier assumptions that filial cannibalism is primarily determined by extrinsic factors such as clutch size, egg quality, or male condition, our study highlights the potentially substantial impact of intrinsic factors as captured in animal personality.

Intriguingly, the activity cannibalism syndrome was apparent only in the first of two consecutive breeding cycles, while all males expressed similar, and rather high, cannibalism levels during the second breeding cycle. Comparable modulations of behavioral types and syndromes by environmental, seasonal, or social contexts are increasingly recognized (reviewed in Dingemanse et al. 2010; Sih et al. 2012). For example, the syndrome linking exploration with activity and aggressiveness in juvenile trout emerged only 2 months after exposure to predation risk (Adriaenssens and Johnsson 2013). In water striders, exploration personality only arose in a social treatment with many interaction partners and not in a non‐social treatment with isolated pairs (Han and Brooks 2014). Similar to the fading differences between activity types documented in our study, differences between personality types (here: fast vs. slow explorers) were prominent only at early ages. Fast males consecutively became slower and ultimately converged with males of the rather constant slow exploration type (Han and Brooks 2014).

Such convergence could arise if the degree of individual plasticity in personality traits (and consequently in any related behavioral syndrome) varied among individuals but correlated with the underlying personality trait. Inherent individual variation in reaction norms has recently been described, for example, in house sparrows (Westneat et al. 2011), where some parents adaptively adjusted provisioning to nestling growth while others did (and likely could) not. Similar differences in reaction norms could explain our finding: Under seasonal progress, high‐active males (perhaps due to limited reaction norms) maintained high levels of FC regardless of breeding cycle, while low‐active males elevated their initially low levels of FC only during the second breeding cycle (Fig. 6).

In the following, we first discuss why FC levels may be generally higher later in the season, covering abiotic factors, breeding system characteristics, and individual characteristics, all of which vary with breeding season. Second, we propose a combination of factors that plausibly affects low‐ and high‐active individuals to a different degree and thus explains why cannibalism rates converge during the second breeding cycle. We furthermore discuss activity differences between contexts and general implications of the link between activity and FC.

While a laboratory study in the closely related sand goby, *Pomatoschistus minutus*, did not find any effect of season on FC (Lissåker 2007), river bullheads, *Cottus gobio*, and mouth breeding cardinal fish, *Apogon doederleini*, increased FC with advancing breeding season (Marconato et al. 1993; Okuda and Yanagisawa 1996). At first sight, such patterns contradict theory, which predicts that FC should be higher at the beginning of the season, as parents can invest the energy gained by offspring consumption into future broods (Rohwer 1978; Sargent 1992). However, in both aforementioned studies, parent condition deteriorated over the season (Marconato et al. 1993; Okuda and Yanagisawa 1996), which corresponds to the notion that FC is dependent on the physical condition of the parent (Rohwer 1978; Sargent 1992). In common gobies, it has been shown that supplementary feeding reduces cannibalism rates (Kvarnemo et al. 1998). Given the general increase in FC over time in our study, it may be that the feeding regime was insufficient to compensate for the high energetic loss due to caring behavior, although we did not measure parent body condition to assess this.

Egg quality may vary over the breeding season, leading to increased or decreased selective FC of inferior eggs (Klug and Lindström 2008). Changes in food availability in the field could, for instance, influence female condition and thus egg size, a potential predictor of egg quality (Brooks et al. 1997; Kamler 2005; Klug and Lindström 2008). However, egg diameter did not influence cannibalism in our study, and other work suggests that egg quality typically rather increases with female age (Brooks et al. 1997; Kamler 2005). Nevertheless, time of the season itself may determine the prospective fitness of newly produced offspring and thus the reproductive value of individual clutches. For instance, early hatching bluegill sunfish larvae, *Lepomis macrochirus*, have a growth advantage and higher survivorship to the following breeding season (Cargnelli and Gross 1996, 1997). If applicable to common gobies, males may preferentially consume the less valuable offspring hatching later in the season.

Alternatively, FC could increase due to a higher mate availability and thus likelihood for males to receive further clutches. Mate availability has been shown to vary with time in two‐spotted gobies, *Gobiusculus flavescens,* where a male‐biased operational sex ratio (OSR) shifted toward a female bias within a single breeding season (Forsgren et al. 2004). We assume a similar change in OSR in field populations of the common goby (K. Heubel, personal observation). Availability of fecund females can accelerate FC (Takeyama et al. 2013) and has been modeled as one determinant of FC, based on the assumption that consumed eggs can more easily be replaced if many gravid females are available for spawning (Kondoh and Okuda 2002). Although mainly applicable to total FC, this prediction may also hold for partial FC in common gobies, as males can accommodate clutches from several females simultaneously and could fill up gaps in their brood with eggs from a new female. It is, however, unclear whether males in a laboratory setting are able to connect time in the year with prospective female availability.

Two of our covariates differed substantially between the two breeding cycles: clutch size and water temperature, both of which were higher in cycle 2 (Fig. 3). Partial filial cannibalism is generally expected to increase with clutch size given that the fitness costs of consuming a single egg diminish with brood size (Rohwer 1978; Manica 2002b). However, FC was independent of clutch size in Experiment 1 and within each of the two breeding cycles (i.e., clutch size centered around cycle means) in Experiment 2. In addition, previous studies in both common goby (Svensson et al. 1998) and sand goby (Forsgren et al. 1996) found negative relationships between clutch size and FC, rendering clear predictions on the effect of clutch size difficult.

Temperature severely affects an animal's metabolism and behavior, especially in aquatic ectotherms (Schmidt‐Nielsen 1997). Elevated temperatures give rise to higher metabolic rates, which typically result in higher activity levels and increased energy demands (Schmidt‐Nielsen 1997; Biro et al. 2007). Remarkably, such temperature related effects on activity and feeding even occur under minor temperature increases (Biro et al. 2007, 2010). Hence, increased energetic requirements due to higher temperatures in the second breeding cycle may have led to the observed elevated cannibalism levels.

While all the above arguments help understanding why FC may generally increase later in the season, none of these explain why this increase only occurred in low‐activity males but not in high‐activity males. Assuming rising water temperatures indeed increased male activity, this may have been true only for low‐activity individuals, while high‐activity males may have already been at their upper activity limit (see Westneat et al. 2011 for a similar scenario on individual upper limits of food provisioning). Due to such limitations on individual reaction norms, activity differences between experimental groups may have become negligible, leading to similar cannibalism levels in the second breeding cycle. As male activity was not measured again after the initial activity scoring, we lack data to further assess this idea. Likewise, individual reaction norms for cannibalism could be limited with high‐activity males already closer to their maximum early in the season. Seasonal trajectories and personality type‐specific life‐history reaction norms could thus have eroded the initial individual differences.

In addition to the correlation between activity and FC, we found activity to be consistently different between individuals during Experiment 1. However, beyond this consistency, we found average activity levels to be clearly lower during breeding, even though they were scored during breaks in male brood care only. Nest holders that are not yet accommodating clutches may still explore their nearby environment to locate even better nesting sites (Magnhagen 1998). When eggs are present, fending off egg predators or conspecific males that may cannibalize the young requires males to stay at least near the nest (Nyman 1953; Magnhagen and Vestergaard 1991). Furthermore, given that egg fanning is energetically costly (Lindström and Hellström 1993) males may partially compensate by being comparably less active in the remaining time during their paternal care phases.

Overall, both experiments revealed a positive relationship between activity and FC, hinting at an intrinsic coupling of both behaviors. However, given that more active males potentially expended more energy, they also may have used their eggs as an additional food source, resulting in higher cannibalism rates. As mentioned before (see Introduction), evidence for such an energy dependence of FC as proposed by Rohwer (1978) remains ambiguous. Our finding that total FC occurred more frequently on smaller clutches in Experiment 2 fits well with previous studies (e.g., Forsgren et al. 1996; Lindström and Sargent 1997; Manica 2002a) and corresponds to the idea that small broods, which are of lower reproductive value, are terminated to reallocate time and energy to prospective future clutches (Rohwer 1978; Sargent 1992). In contrast, total FC in Experiment 1 increased with activity. While more brood abortions combined with higher activity would in principle also conform with the energy‐based hypothesis as the cost of care may get too high (Manica 2002b), this is not supported by a feeding experiment in common gobies, where males given food in excess did not change TFC (Kvarnemo et al. 1998). In our study, where males experienced no food shortage, we consider it unlikely that the relationship between activity and FC can be adequately explained solely by energetic requirements.

Although we cannot rule out the possibility that cannibalism was at least partly driven by energetic needs, and despite indications that individual reactions norms in response to environmental variation may vary among personality types (Dingemanse et al. 2010; Westneat et al. 2011), our data appear most consistent with the idea that activity and FC form a personality‐mediated behavioral syndrome, implying that these traits cannot be adjusted fully independently of each other. General activity could be seen as a favorable trait, as more activity presumably leads to more foraging success (Toscano and Griffen 2014), while inappropriately high levels of cannibalism should compromise reproductive output. Accordingly, animal personality could limit an individual's ability to adapt to the requirements of paternal care and may thus have important fitness consequences. Following this reasoning, filial cannibalism may be partly driven by negative spillover effects due to an intrinsic linkage with activity (Johnson and Sih 2005). One implication of this finding would be that male activity could serve common goby females as a criterion to discriminate against particularly active males and the associated high risk that their eggs become cannibalized. Ongoing studies address corresponding female choice of certain personality types (N. Kalb et al, unpubl. ms.).

To conclude, we have shown that filial cannibalism in common gobies is connected to activity, a behavior with consistent individual differences. While we cannot rule out an energy‐based explanation, available evidence supports an intrinsic coupling of both traits. At the same time, FC seems to be influenced by the progressing breeding season with its multitude of varying external factors. Although behavioral plasticity is likely restricted through animal personality, cannibalism is thus nevertheless heavily affected by environmental conditions. Future research is crucial to further our understanding not only of fitness consequences of personality‐mediated filial cannibalism, but also of the underlying interplay of behavioral syndromes and environmental variation.

## Conflict of Interest

The authors declare that they have no conflict of interest.

## Data accessibility

Data available from the Dryad Digital Repository: http://dx.doi.org/10.5061/dryad.q8k52.
